# Genetic Fusions of a CFA/I/II/IV MEFA (Multiepitope Fusion Antigen) and a Toxoid Fusion of Heat-Stable Toxin (STa) and Heat-Labile Toxin (LT) of Enterotoxigenic *Escherichia coli* (ETEC) Retain Broad Anti-CFA and Antitoxin Antigenicity

**DOI:** 10.1371/journal.pone.0121623

**Published:** 2015-03-24

**Authors:** Xiaosai Ruan, David A. Sack, Weiping Zhang

**Affiliations:** 1 Department of Diagnostic Medicine/Pathobiology, Kansas State University College of Veterinary Medicine, Manhattan, KS, 66506, United States of America; 2 Department of International Health, Johns Hopkins University, Bloomberg School of Public Health, Baltimore, MD, 21205, United States of America; University of Minnesota, UNITED STATES

## Abstract

Immunological heterogeneity has long been the major challenge in developing broadly effective vaccines to protect humans and animals against bacterial and viral infections. Enterotoxigenic *Escherichia coli* (ETEC) strains, the leading bacterial cause of diarrhea in humans, express at least 23 immunologically different colonization factor antigens (CFAs) and two distinct enterotoxins [heat-labile toxin (LT) and heat-stable toxin type Ib (STa or hSTa)]. ETEC strains expressing any one or two CFAs and either toxin cause diarrhea, therefore vaccines inducing broad immunity against a majority of CFAs, if not all, and both toxins are expected to be effective against ETEC. In this study, we applied the multiepitope fusion antigen (MEFA) strategy to construct ETEC antigens and examined antigens for broad anti-CFA and antitoxin immunogenicity. CFA MEFA CFA/I/II/IV [CVI 2014, 21(2):243-9], which carried epitopes of seven CFAs [CFA/I, CFA/II (CS1, CS2, CS3), CFA/IV (CS4, CS5, CS6)] expressed by the most prevalent and virulent ETEC strains, was genetically fused to LT-STa toxoid fusion monomer 3xSTa_A14Q_-dmLT or 3xSTa_N12S_-dmLT [IAI 2014, 82(5):1823-32] for CFA/I/II/IV-STa_A14Q_-dmLT and CFA/I/II/IV-STa_N12S_-dmLT MEFAs. Mice intraperitoneally immunized with either CFA/I/II/IV-STa_-toxoid_-dmLT MEFA developed antibodies specific to seven CFAs and both toxins, at levels equivalent or comparable to those induced from co-administration of the CFA/I/II/IV MEFA and toxoid fusion 3xSTa_N12S_-dmLT. Moreover, induced antibodies showed *in vitro* adherence inhibition activities against ETEC or *E*. *coli* strains expressing these seven CFAs and neutralization activities against both toxins. These results indicated CFA/I/II/IV-STa_-toxoid_-dmLT MEFA or CFA/I/II/IV MEFA combined with 3xSTa_N12S_-dmLT induced broadly protective anti-CFA and antitoxin immunity, and suggested their potential application in broadly effective ETEC vaccine development. This MEFA strategy may be generally used in multivalent vaccine development.

## Introduction

Virulence heterogeneity among bacterial and viral strains or isolates has long been one major challenge in vaccine development. Like many other infectious pathogens, enterotoxigenic *Escherichia coli* (ETEC) strains (i.e., *E*. *coli* producing heat-labile and heat-stable enterotoxins) are immunologically heterogeneous. ETEC strains are the most common bacterial cause of diarrhea which continues to be the second leading cause of death in children younger than 5 years who live in developing countries and remains a major threat to global health [1,2]. These ETEC strains express immunologically different colonization factor antigen (CFA) adhesins and enterotoxins. CFA adhesins mediate bacterial attachment to host receptors and facilitate colonization in host small intestines. Enterotoxins disrupt fluid and electrolyte homeostasis in host small intestinal epithelial cells that leads to fluid hyper-secretion and diarrhea [3]. Therefore, CFA adhesins and enterotoxins are recognized the major virulence determinants. There are at least 23 CFA or CS (coli surface antigen) adhesins and two very distinct enterotoxins characterized among ETEC strains isolated from humans with diarrhea [3–6]. Enterotoxins produced by ETEC strains associated with human diarrhea are heat-labile toxin (LT) and heat-stable toxin type Ib (STa, human-type STa, STh or hSTa). Although heat-stable toxin type Ia (porcine-type STa, STp or pSTa) and heat-stable toxin type II (STb), together with LT and/or STa, are occasionally detected in ETEC strains isolated from human diarrheal patients, pSTa and STb toxins cause diarrhea only in animals [3]. Since ETEC strains expressing any one or two CFA or CS adhesins and LT or STa enterotoxin cause diarrhea, developing broadly protective vaccines against ETEC diarrhea continues to be very challenging [7,8].

Anti-CFA antibodies specific to an individual CFA or antitoxin antibodies to LT were reported to provide protection to human volunteers against homologous challenge [9–12], but not against ETEC strains expressing immunologically heterogeneous CFAs or the STa toxin. Early experimental vaccine studies showed that killed ETEC prototype strain H10407 (O78:H11, LT^+^STa^+^CFA/I^+^) induced anti-CFA/I and anti-LT antibodies that protected volunteers against challenge of strain H10407 or a homologous strain [13,14]. These observations led to a conceptual ETEC vaccine, that is, an ETEC vaccine that should induce antibodies protecting against multiple CFA adhesins and toxin LT [15]. Consequently, products that were the result of mixing together a few strains that expressed several CFA adhesins and recombinant LT-B subunit protein or the homologous cholera toxin B subunit (CT-B) were examined for protection against ETEC diarrhea. Experimental vaccines currently under development include two oral whole-cell ETEC vaccine candidates, rCTB-CF and ACE527. The rCTB-CF consists of five killed strains expressing six CFA adhesins plus recombinant CT-B subunit protein [16,17], and the ACE527 is composed of three live attenuated *E*. *coli* strains that express five CFA adhesins, one CFA subunit, and LT-B subunit [18,19]. Recombinant CT-B subunit was included in the rCTB-CF product because anti-CT-B antibodies were shown to provide short-term protection against LT-producing ETEC strains [20]. Field studies showed that the rCTB-CF vaccine induced antibody responses and protected adults traveling from developed countries to ETEC endemic countries against the risk of disease by 60% to 70% [16,17] or against moderate to severe diarrhea [21]. This product, however, provided no protection to children, especially very young children living in endemic areas against ETEC diarrhea, and caused some adverse effects in very young children when an adult dose was given orally [22,23]. In addition, it did not reduce the overall diarrhea rate among US adults traveling to Guatemala and Mexico [21]. The live ACE527 product was found associated with some adverse effects in volunteer studies, but the adverse effects were reduced or eliminated when a lower dose was given [24]. This ACE527 induced antibody responses to LT-B, CFA/I, CS3, and CS6 among adult volunteers, but protected against only the severity of diarrhea outcome from homologous challenge [18,25].

Although efforts have been undertaken to continuously improve current vaccine candidates [24,26,27], these products might still be unable to provide truly broad protection against ETEC diarrhea because they carry only LT-B or CT-B subunit antigen but no STa antigens to induce antibodies against STa toxin. Anti-LT antibodies were found protective against ETEC strains expressing LT but not against ETEC strains expressing the heat-stable toxin [28,29]. Furthermore, cocktail products require a relatively high oral dose to stimulate host immune responses against individual CFA adhesins and LT. A high oral dose delivers many somatic antigens, particularly lipopolysaccharide (LPS) which is thought to be the cause of the gastrointestinal side effects associated with these vaccine candidates, especially in young children [30], and may also mask stimulation of host anti-CFA and anti-LT (or anti-CT) specific antibody responses [8,31].

Ideally, a vaccine should induce antibodies against all CFA adhesins and both toxins, particularly STa toxin. In fact ETEC strains producing STa as the only toxin are the frequent cause of ETEC diarrhea in developing countries [32]. ETEC strains that produce STa with or without LT are associated with over two-thirds of human ETEC diarrhea cases [5], and tend to cause moderate to severe diarrhea more frequently [21,33]. Therefore, ETEC vaccines may also need to induce protective anti-STa antibodies in order to provide broad protection against ETEC diarrhea.

Although STa is poorly immunogenic because of its small size (19 amino acids), it is a very potent enterotoxin. Thus, STa presents a problem for use as a potential immunogen because it does not induce immunity following exposure and also causes disease unless inactivated [34,35]. However, recent studies demonstrated that STa, when had a single amino acid substituted, was less or non-toxic; moreover, derived STa toxoids became immunogenic after being genetically fused to a strongly immunogenic LT toxoid monomer (a single peptide consisting of a mutated LT-A subunit and one LT-B subunit) [36,37]. LT-STa toxoid fusions that carried different STa toxoids were found to induce neutralizing antibodies against both LT and STa toxins [38].

To induce anti-CFA antibodies effectively protecting against heterogeneous CFA adhesins is another challenge in ETEC vaccine development. A recent study showed that a single CFA/I/II/IV multiepitope fusion antigen (MEFA) was constructed and this CFA/I/II/IV MEFA induced antibodies cross protective against seven ETEC CFA adhesins, CFA/I, CFA/II (CS1, CS2, CS3) and CFA/IV (CS4, CS5, CS6) [39]. However, although these seven CFA adhesins are expressed by ETEC strains causing 70–80% of ETEC diarrhea cases [23], their prevalence varies greatly at different geographic locations [40]. Thus, antibodies against these seven CFA adhesins may still not be sufficient to effectively protect against ETEC diarrhea in some regions.

In contrast to CFA adhesins with variable prevalence geographically, LT and STa, alone or together, are expressed by all ETEC strains that cause diarrhea in humans. But anti-toxin antibodies without assistance from antibodies against CFA adhesins may be less effective against ETEC diarrhea [41]. If the CFA/I/II/IV MEFA is combined with or further fused to an optimal LT-STa toxoid fusion that induces protective antibodies against both toxins, the resultant product should be able to induce immunity to provide even broader protection against ETEC diarrhea, since the antitoxin antibodies provide supplementary protection against ETEC strains expressing these seven CFA adhesins and independent protection against the ETEC strains expressing the other CFA adhesins.

To create a single antigen inducing antibodies broadly protecting against seven CFA adhesins and both toxins and to explore potential application of such an antigen in ETEC vaccine development, in this study we first genetically fused the CFA/I/II/IV MEFA gene [39] to a LT-STa toxoid fusion gene [38] for a CFA/I/II/IV-STa_-toxoid_-dmLT MEFA and examined anti-CFA and antitoxin immunogenicity in a murine model. We then evaluated the induced antibodies for neutralization activities against adherence from these seven CFA adhesins and enterotoxicity of both toxins. In addition, we immunized mice with the CFA/I/II/IV MEFA combined with toxoid fusion 3xSTa_N12S_-dmLT, and compared induced antigen-specific antibody responses with those induced by the CFA/I/II/IV-STa_N12S_-dmLT MEFA to assess whether genetic fusion affected antigenic property of the fused CFA adhesin and toxin antigen components, thus enabling application of MEFA strategy for multivalent vaccine development.

## Materials and Methods

### Bacterial strains and plasmids

The *E*. *coli* strains and plasmids used in this study are listed in Table 1. ETEC strains deposited at Johns Hopkins University, Washington University and the *E*. *coli* Reference Strain Center at University of Gothenburg (Sweden), and two recombinant *E*. *coli* strains expressing CS1 and CS2 adhesins (gifts from Dr. J. Scott at Emory University) [42,43] were used for CFA adhesin extraction and in antibody adherence inhibition assays. CFA and CS fimbriae and non-fimbrial outer membrane proteins were generally referred to as CFA adhesins in this study, and LT, STa, and derived toxoids described were of human-type. Recombinant strains 9175 (CFA/I/II/IV MEFA) [39] and 9164 (3xSTa_A14Q_-tmLT_S63K/R192G/L211A_) [37] were used as templates first to construct the CFA/I/II/VI-STa_A14Q_-dmLT MEFA gene. Recombinant strain 9318 (3xSTa_N12S_-dmLT_R192G/L211A_) was included for the construction of CFA/I/II/VI-STa_N12S_-dmLT MEFA, after 3xSTa_N12S_-dmLT was identified as the optimal LT-STa toxoid fusion in inducing anti-STa antibody response [38]. *E*. *coli* BL21 (GE Healthcare, Piscataway, NJ) and vector pET28α (Novagen, Madison, WI) were used to express the CFA/I/II/VI-STa_-toxoid_-dmLT MEFA proteins.

**Table 1:** *Escherichia coli* strains and plasmids used in the study.

Strains	Relevant properties	Sources
BL21	*F* ^*-*^ *omp*T *hsd*S (r_B_^-^, m_B_^-^), *gal dcm*.	GE Healthcare
H10407	O78:H11; CFA/I, LT, STa	Johns Hopkins Univ.
UM 75688	CS5/CS6, LT, STa	Johns Hopkins Univ.
E106 (E11881/9)	CS4/CS6, LT, STa	Univ. of Gothenburg
E116 (E19446)	CS3, LT, STa	Univ. of Gothenburg
2423. ETP98066	CS6, LT, STa	Washington Univ.
THK38/pEU405	CS1	Emory Univ.
DH5α/pEU588	CS2	Emory Univ.
9175	pCFA/I/II/IV in BL21	[39]
9164	p3xSTa_A14Q_-tmLT in BL21	[37]
9318	p3xSTa_N12S_-dmLT in BL21	[38]
9208	pCFA/I/II/IV-STa_A14Q_-dmLT in BL21	this study
9401	pCFA/I/II/IV-STa_N12S_-dmLT in BL21	this study
Plasmids		
pET28α		Novagen
pEU405	CS1	Emory Univ.
pEU588	CS2	Emory Univ.
pCFA/I/II/IV	CFA MEFA subunit gene in pET28α at NheI/EagI	[39]
p3xSTa_A14Q_-tmLT	3xSTa_A14Q_-tmLT fusion gene in pET28α at NheI/EagI	[37]
p3xSTa_N12S_-dmLT	3xSTa_N12S_-dmLT fusion gene in pET28α at NheI/EagI	[38]
pCFA/I/II/IV-STa_A14Q_-dmLT	CFA/I/II/IV-STa_A14Q_-dmLT MEFA in pET28α at NheI/EagI	this study
pCFA/I/II/IV-STa_N12S_-dmLT	CFA/I/II/IV-STa_N12S_-dmLT MEFA in pET28α at NheI/EagI	this study

### CFA/I/II/IV-STa_-toxoid_-dmLT MEFA gene construction

**Fig 1:**
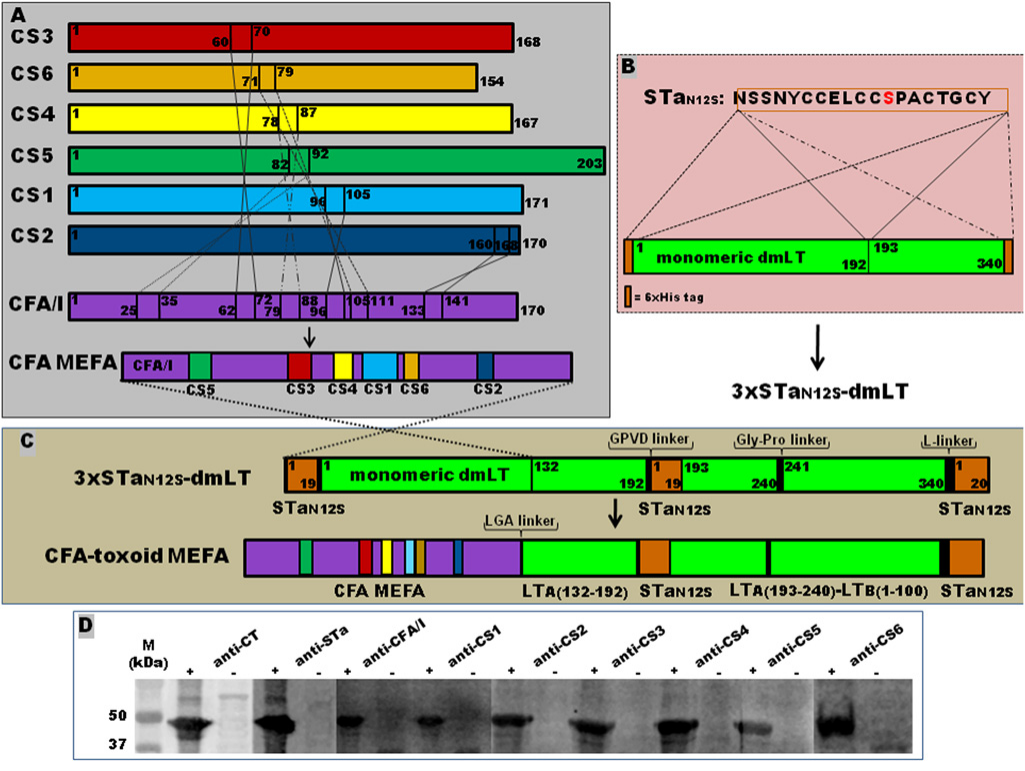
Construction and detection of the CFA/I/II/IV-STa_-toxoid_-dmLT MEFA. (A) Construction of the CFA/I/II/IV MEFA. The most antigenic epitopes of the CS1, CS2, CS3, CS4, CS5 and CS6 major structural subunits were embedded into CFA/I major subunit by replacing the CfaB surface-exposed but less antigenic epitopes. (B) Construction of the 3xSTa_N12S_-dmLT toxoid fusion. Three copies of the STa toxoid STa_N12S_ gene were genetically fused to the monomeric dmLT (LT_R192G/L211A_) gene using SOE (splicing overlap extension) PCRs. (C) Construction of CFA/I/II/IV-STa_N12S_-dmLT MEFA. A substitution of the first 150 amino acids of the 3xSTa_N12S_-dmLT (the N-terminal STa_N12S_ and the first 131 amino acids of LT-A subunit) with the CFA/I/II/IV MEFA created the CFA/I/II/IV-STa_N12S_-dmLT MEFA. Four linkers: LGA, GPVD, Gly-Pro linker GPGP, and L-linker were used for the construction. (D) Western blot to detect the CFA/I/II/IV-STa_N12S_-dmLT MEFA protein with anti-CFA/I, anti-CS1, -CS2, -CS3, -CS4, -CS5, and anti-CS6 MAb hybridoma supernatant (1:100; provided by Dr. AM Svennerholm), and rabbit anti-CT (1:3300; Sigma) and anti-STa antiserum (1:3300; provided by Dr. DC Robertson). Extracted MEFA proteins separated in 12% PAGE gel were detected with each anti-adhesin MAb, anti-CT and anti-STa antiserum and IRDye-labeled goat anti-mouse IgG or anti-rabbit IgG (1:5000; LI-COR). Lane (+) indicated the CFA/I/II/IV-STa_N12S_-dmLT MEFA proteins, whereas lane (-) of extracted total proteins of *E*. *coli* BL21 host strain as the negative control. Lane M is the protein marker (in kilo Daltons; Precision Plus Protein pre-stained standards; Bio-Rad).

Splicing overlap extension (SOE) PCR was used to construct CFA/I/II/IV-STa_-toxoid_-dmLT MEFA genes as described previously [34,37]. Briefly, two PCR products, the multiepitope CFA/I/II/IV fragment and the STa_-toxoid_-dmLT toxoid fusion fragment, were overlapped for a single open-reading-framed CFA/I/II/IV-STa_-toxoid_-dmLT chimeric gene (Fig. 1). The multiepitope CFA/I/II/IV fragment, which carried antigenic epitopes of seven CFA adhesins (CFA/I, CFA/II, and CFA/IV), was amplified in a PCR with primers T7-F (5’-TAATACGACTCACTATAGGG-‘3) and CFA-toxoid-R (5’- ACCAAAGGCTCCCAAAGTCATTACAAGAGATACTACTCCTGA-‘3) using plasmid pCFA/I/II/IV [39] as the DNA template. The STa_-toxoid_-dmLT toxoid fragment, which consisted of 2 copies of the STa_-toxoid_ (between the LT-A1 and LT-A2 peptides and at the LT-B C-terminus), dmLT-A (132–240 amino acids) and LT-B, was amplified with primers CFA-toxoid-F (5’-GTAATGACTTTGGGAGCCTTTGGTGTGATTGATGAACGATTACATCGT-‘3) and T7-R (5’-TGCTAGTTATTGGTCAGGGGT-‘3). Two different toxoid fusion fragments were generated by using plasmid p3xSTa_A14Q_-tmLT [37] and plasmid p3xSTa_N12S_-dmLT [38] as the DNA template, respectively. Each amplified toxoid fusion fragment was overlapped to the multiepitope CFA/I/II/IV fragment to generate CFA/I/II/IV-STa_A14Q_-dmLT and CFA/I/II/IV-STa_N12S_-dmLT. CFA/I/II/IV-STa_A14Q_-dmLT and CFA/I/II/IV-STa_N12S_-dmLT were further PCR amplified with primers T7-F and T7-R. Amplified products were digested with NheI and EagI restriction enzymes (New England BioLabs, Ipswich, MA) and ligated into expression vector pET28α. Cloned CFA/I/II/IV-STa_-toxoid_-dmLT chimeric genes were verified with DNA sequencing.

### Expression, extraction and detection of CFA/I/II/IV-STa_-toxoid_-dmLT MEFA proteins

*E*. *coli* strain BL21 was used to host plasmid carrying CFA/I/II/IV-STa_A14Q_-dmLT or CFA/I/II/IV-STa_N12S_-dmLT chimeric gene to express two CFA/I/II/IV-STa_-toxoid_-dmLT MEFA proteins. Each recombinant strain (from a single colony) was cultured in 5 ml Luria Bertani (LB) broth supplemented with kanamycin (30μg/ml) at 37°C on a shaker (150 rpm). Overnight grown culture was added to 500 ml 2x YT (2x Yeast Extract Tryptone) medium broth for continued incubation. Bacteria were induced with isopropyl-1-thio-β-D-galactoside (IPTG; 0.5 mM) after culture optical density reached 0.5 at 600 nm (OD_600_), and were incubated for 4 more hours. Bacterial culture was centrifuged at 5,000 X g for 20 min, and pellets were used to extract total insoluble proteins (inclusion body fraction) using bacterial protein extraction reagent (B-PER, in phosphate buffer; Pierce, Rockford, IL) by following the manufacturer’s protocol.

Recombinant CFA/I/II/IV-STa_-toxoid_-dmLT MEFA proteins tagged with six histidines were further extracted to a purity of greater than 90% using Ni-nitrilotriacetic acid (NTA) agarose (QIAGEN, Valencia, CA). 6xHis-tagged fusion proteins were refolded using a Protein Refolding kit by following the manufacturer’s protocols (Novagen, Madison, WI). Refolded 6xHis-tagged proteins were dialyzed in 20 mM Tris-HCl buffer overnight at 4°C, and were concentrated (to 1–2 mg/ml) using Spectra/Por molecularporous membrane tubing (Spectrum Laboratories Inc., Rancho Dominquez, CA) and polyethylene glycol compound (PEG; Sigma, St. Louis, MO) as described previously [37,44].

Ten microliter each refolded CFA/I/II/IV-STa_-toxoid_-dmLT MEFA protein (10–20 μg) was analyzed in 12% sodium dodecyl sulfate polyacrylamide gel electrophoresis (SDS-PAGE) and immune blot assays. Mouse anti-CFA/I, anti-CS1, anti-CS2, anti-CS3, anti-CS4, anti-CS5 and anti-CS6 monoclonal antibody hybridoma supernatant (in dilution of 1:100; provided by Dr. AM Svennerholm) [39], rabbit anti-CT serum (1:3300; Sigma), and protein-A column-purified rabbit anti-STa serum (1:3300; provided by Dr. DC Robertson) [34] were used as the primary antibody. IRDye-labeled goat anti-mouse or anti-rabbit IgG (1:5000; LI-COR, Lincoln, NE) was used as the secondary antibody. CFA/I/II/IV-STa_-toxoid_-dmLT MEFA proteins were detected with a LI-COR Odyssey premium infrared gel imaging system (LI-COR).

### Mouse immunization with CFA/I/II/IV-STa_-toxoid_-dmLT MEFA proteins

Mouse immunization studies complied with the Animal Welfare Act by following the 1996 National Research Council guidelines [45], and were approved and supervised by a state veterinarian and the Kansas State University Institutional Animal Care and Use Committee. Data generated from this study are publicly available. Before being used in mouse immunization, CFA/I/II/IV-STa_-toxoid_-dmLT MEFA proteins were verified non-toxic using T-84 cells and EIA cAMP and cGMP kits (Assay Design, MI) as previously described [34,37,38].

Four groups of 6- to 8-week-old female BALB/c mice (Charles River Laboratories International, Inc., Wilmington, MA) were included in the immunization study. The first group of 8 mice was each injected intraperitoneally (i.p.) with 200 μg refolded CFA/I/II/IV-STa_A14Q_-dmLT MEFA protein (in 200 μl 20 mM Tris-HCl) and 200 μl Freund’s complete adjuvant (FCA; Sigma). The second group of 15 mice was each injected (i.p.) with 200 μg refolded CFA/I/II/IV-STa_N12S_-dmLT MEFA protein (in 200 μl 20 mM Tris-HCl) with 200 μl FCA. The third group of 16 mice was each co-administered (i.p.) with refolded CFA/I/II/IV MEFA protein and 3xSTa_N12S_-dmLT toxoid fusion protein produced previously [38,39]. To keep the molecule copy numbers of CFA/I/II/IV and 3xSTa_N12S_-dmLT antigens equivalent to 200 μg CFA/I/II/IV-STa_N12S_-dmLT (based on peptide lengths of the CFA/I/II/IV and the STa_N12S_-dmLT peptides), 80 μg CFA/I/II/IV protein and 150 μg 3xSTa_N12S_-dmLT protein were mixed (in a total of 200 μl 20 mM Tris-HCl) and used (with 200 μl FCA) to immunize each mouse in this co-administration group. The fourth group of 9 mice was each injected (i.p.) with 200 μl Freund’s complete adjuvant and 200 μl protein buffer 20 mM Tris-HCl as the negative control. Two booster injections at the same doses as the primary immunization but with Freund’s incomplete adjuvant (FIA) were followed at a bi-week interval. In addition, a group of 5 mice was each immunized (i.p.) with 200 μg CFA/I fimbriae which were heat-extracted from ETEC H10407 and 200 μl Freund’s adjuvants (FCA in the primary and FIA in boosters) as the positive control for mouse anti-CFA/I antibody response.

Blood samples were collected from each mouse before the primary immunization and 10 to 12 days after each immunization to prepare serum samples. Serum samples were stored at -80°C until use. On day 37 post primary immunization, mice were anesthetized with CO_2_ and exsanguinated.

### Mouse anti-CFA and anti-toxin antibody titration

Anti-CFA/I and anti-CS1, -CS2, -CS3, -CS4/CS6, -CS5/CS6, and anti-STa and anti-LT IgG antibodies in serum of each mouse were titrated as described previously [34,37–39]. Briefly, 500 ng CFA/I, CS1, CS2, CS3, CS4/CS6, and CS5/CS6 heat-extracted from ETEC field isolates or recombinant *E*. *coli* strains [39] (Table 1), or 100 ng LT (List Biological Laboratories, Inc., Campbell, CA) were coated to each well of H2B plates (Thermo Scientific, Rochester, NY) to titrate antibodies specific to each CFA and LT; whereas 10 ng STa-ovalbumin conjugates were coated to each well of Costar plates (Corning Inc., Corning, NY) to titrate anti-STa IgG antibodies. All serum samples were examined in triplicate. Horseradish peroxidase (HRP)-conjugated goat anti-mouse IgG (1:3300; Sigma) and 3,3’,5,5’-tetramethylbenzidine (TMB) Microwell Peroxidase Substrate System (2-C) (KPL, Gaithersburg, MD) were used to measure optical density (OD) at the wavelength of 405 nm. Antibody titers were calculated from the highest dilution of a serum sample that produced OD readings of > 0.3 above the background readings and were shown in a log_10_ scale as previously described [36,38].

### Anti-CFA antibody adherence inhibition assay

Mouse serum samples pooled from each group were examined for *in vitro* antibody adherence inhibition activity against ETEC strains expressing CFA/I, CS3, CS4/CS6, CS5/CS6, or CS6 and *E*. *coli* recombinant strains expressing CS1 or CS2, using Caco-2 cells (ATCC, #HTB-37) as previously described [39]. ETEC or *E*. *coli* bacteria expressing CFA/I, CFA/II or CFA/IV adhere to Caco-2 cells, whereas neutralizing anti-CFA antibodies in mouse serum samples block adherence of these ETEC or *E*. *coli* bacteria to Caco-2 cells. Therefore, by mixing the mouse serum and bacteria of each strain, incubating the mixture with Caco-2 cells, and counting the bacteria adhered to the incubated cells, we were able to measure serum antibody adherence inhibition activities against ETEC or *E*. *coli* expressing these seven CFA adhesins. Briefly, Caco-2 cells were seeded and grown in 75 ml flask (Corning). After growth of confluence, Caco-2 cells were transferred to each well of a 12-well tissue culture plate containing Dulbecco’s modified Eagle’s medium (DMEM)-20% fetal bovine serum (FBS) (Fisher Thermo Scientific, Pittsburg, PA), and grown to a confluent monolayer (7x10^5^ per well). ETEC and *E*. *coli* bacteria, after overnight growth on sheep blood agar plates at 37°C, were scraped off with cotton swabs and were gently suspended in sterile PBS. One hundred microliters of each bacterial suspension (3.5x10^6^ bacteria; with a multiplicity-of-infection ratio set at five bacteria to one Caco-2 cell) were incubated with 20 μl serum sample pooled from the mice in each group on a shaker (50 rpm) for 1 h at room temperature. The bacteria and serum mixture was brought to 300 μl with PBS, and was added to each well containing the Caco-2 cells (in 700 μl cell culture medium). After incubation in a CO_2_ incubator (5% CO_2_) for 1 h at 37°C, wells were gently washed with PBS to remove non-adherent ETEC or *E*. *coli* bacteria. Washed Caco-2 cells were dislodged through incubation with 0.25% trypsin (200 μl per well) in a CO_2_ incubator for 30 min at 37°C. Dislodged Caco-2 cells (with adherent ETEC or *E*. *coli* bacteria) were collected by centrifugation (15,000 g for 10 min) and then suspended in 1 ml PBS. Suspensions were gently but well mixed, serially diluted, and plated on LB plates. After overnight growth at 37°C, ETEC or *E*. *coli* bacteria colony forming units (CFUs) were counted.

### Anti-LT and anti-STa antibody neutralization assays

Serum samples pooled from mice in each group were also examined for *in vitro* antibody neutralization activities against STa and CT using EIA cAMP and cGMP kits (Assay Design) and T-84 cells. STa stimulates an increase of intracellular cyclic GMP levels and CT elevates intracellular cAMP levels in T-84 cells. Neutralizing antitoxin antibodies neutralize enterotoxicity thus prevent STa and CT from stimulating intracellular cGMP or cAMP. Therefore, by incubating serum with the toxin, adding the mixture to T-84 cells, and measuring cGMP or cAMP levels in the cells, we were able to evaluate neutralization activities of mouse serum IgG antibodies against STa or CT. As described previously [34,36–38], 30 μl pooled serum sample of each immunization group or the control group was incubated with 2 ng STa toxin or 10 ng CT for 30 min at room temperature, and the serum/toxin mixture was added to T-84 cells. After incubation in a CO_2_ incubator for 1 h (for STa to measure cGMP) or 3 h (for CT to measure cAMP), T-84 cells were measured for intracellular cGMP or cAMP levels (pmol/ml) with EIA cGMP or cAMP kit by following the manufacturer’s protocol (Assay Design). STa or CT alone (without serum) was used as the control to show enterotoxicity in stimulation of cGMP or cAMP in T-84 cells, and culture medium only (without toxin or serum) was used to show a baseline of intracellular cAMP or cGMP level in T-84 cells.

### Statistical analysis

Data were analyzed using SAS for Windows, version 8 (SAS Institute, Cary, NC). Results were presented as means ± the standard deviations. A Student’s *t*-test was used to compare different treatment groups. Calculated p values of less than 0.05 were considered as significant when treatment groups were compared using two-tailed distribution and two-sample unequal variance. In addition, the non-parametric Mood’s Median Test was carried out to assess differences of antibody neutralization activities (cAMP and cGMP, pmol/ml) from serum samples of the immunized group and the control group.

## Results and Discussion

### Constructed CFA/I/II/IV-STa_-toxoid_-dmLT MEFAs consisted of epitopes of the seven CFA adhesins, two copies of the STa toxoid, and a part of the dmLT monomer

The overlap of CFA/I/II/IV MEFA and the 3xSTa_A14Q_-dmLT or 3xSTa_N12S_-dmLT PCR amplified products yielded two CFA/I/II/IV-STa_-toxoid_-dmLT chimeric genes (Fig. 1). Two recombinant strains, 9208 and 9401, were constructed to express two CFA/I/II/IV-STa_-toxoid_-dmLT MEFA proteins. These two MEFA proteins differed only at the STa toxoid, with STa_A14Q_ in 9208 and STa_N12S_ in 9401 (Table 1). DNA sequencing showed each chimeric gene was a single open reading frame coding a single 6xHis-tagged CFA/I/II/IV-STa_-toxoid_-dmLT protein. Each fusion protein consisted of 20 amino acids from the pET28α vector including the 6xHis tag (six histidines), the CFA/I/II/IV MEFA (150 amino acids) carrying epitopes of CFA/I and CS1-CS6 major subunits (CfaB, CooA, CotA, CstH, CsaB, CsfA and CssA), two copies of STa toxoid STa_A14Q_ or STa_N12S_, 109 amino acids of dmLT-A subunit (132–240, at the C-terminus; with R192G and L211A mutations) and one copy of the LT-B subunit (100 amino acids), and four intra-peptide linkers (Fig. 1C). The first copy of the STa toxoid (without the stop codon) with a ‘GPVD’ linker was located after the mutated 192th amino acid residue (Arg→Gly) of the LT-A subunit, and the second STa toxoid (with the stop codon) with an L-linker was at the C-terminus of the fusion protein.

Coomassie blue stained SDS-PAGE showed over 90% of the proteins extracted from strains 9208 and 9401 had a molecular mass of about 48 KDa, the expected size of the 6xHis-tagged CFA/I/II/IV-STa_-toxoid_-dmLT MEFA protein. This 6xHis-tagged protein was recognized by anti-CFA/I and anti-CS1, -CS2, -CS3, -CS4, -CS5, and anti-CS6 MAb hybridoma supernatant, and rabbit anti-STa and anti-CT sera in Western blot assays (Fig. 1D).

### CFA/I/II/IV-STa_-toxoid_-dmLT MEFA proteins were well tolerated and immunogenic

T-84 cells incubated with 100 μg refolded fusion protein CFA/I/II/IV-STa_A14Q_-dmLT or CFA/I/II/IV-STa_N12S_-dmLT showed no increase of intracellular cAMP and cGMP levels. That indicated neither MEFA protein possessed detectable LT or STa enterotoxicity. In addition, female adult mice did not display any noticeable adverse effects after i.p. immunization with either CFA/I/II/IV-STa_-toxoid_-dmLT MEFA protein. Mice co-administered with the CFA/I/II/IV MEFA protein and the 3xSTa_N12S_-dmLT toxoid fusion protein remained healthy and acted normally.

**Fig 2:**
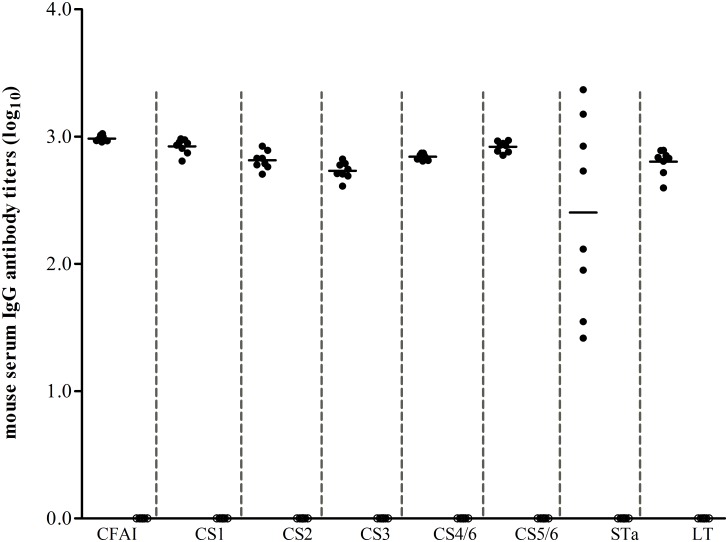
Mouse serum anti-adhesin and antitoxin IgG antibody titers. Anti-CFA/I, anti-CS1, -CS2, -CS3, -CS4/CS6 and anti-CS5/CS6, and anti-STa and anti-LT IgG antibodies in the serum of each mouse immunized with CFA/I/II/IV-STa_A14Q_-dmLT MEFA protein (●) and the serum of each control mouse (○) were titrated in ELISAs. CFA/I, CS1, CS2, CS3, CS4/CS6, CS5/CS6 heat-extracted from *E*. *coli* or ETEC strains in Table 1 (500 ng per well of a 2HB plate), STa-ovalbumin (10 ng per well of a Costar plate), or LT (List Biological Laboratories, Inc.; 100 ng per well of a 2HB plate) and HRP-conjugated goat-anti-mouse IgG (1:3300; the secondary antibodies) were used to titrate IgG antibodies specific to CFA/I, CS1, CS2, CS3, CS4/6, CS5/6 and to STa and LT toxins, respectively. The antibody titer was calculated from the highest dilution of a serum sample that produced an ELISA optical density of greater than 0.3 (above the background) and presented in a log_10_ scale. Each dot represented a mouse IgG titer, and the bars indicated the mean titer of the group.

Mice immunized with CFA/I/II/IV-STa_A14Q_-dmLT or CFA/I/II/IV-STa_N12S_-dmLT developed immune responses to CFA/I, CS1, CS2, CS3, CS4/6, CS5/6, STa, and LT. Serum samples of the mice immunized with fusion CFA/I/II/IV-STa_A14Q_-dmLT had anti-CFA/I, -CS1, -CS2, -CS3, -CS4/6, -CS5/6, -STa, and anti-LT IgG titers (in log_10_) of 2.98±0.02, 2.92±0.06, 2.81±0.07, 2.73±0.07, 2.84±0.03, 2.92±0.04, 2.40±0.75, and 3.19±0.01, respectively (Fig. 2). Serum samples from individual mice immunized with fusion CFA/I/II/IV-STa_N12S_-dmLT also developed IgG antibody responses to CFA/I, CS1, CS2, CS3, CS4/6 and CS5/6 adhesins and both toxins (Table 2). Serum of the mice i.p. immunized with the heat-extracted CFA/I fimbriae had anti-CFA/I IgG titer of 2.95 ±0.01 (log_10_).

**Table 2:** Anti-CFA/I, -CS1, -CS2, -CS3, -CS4/CS5, and anti-CS5/CS6, and anti-STa and anti-LT IgG antibody titers (in log_10_; mean ± standard deviation) detected in the serum of mice immunized with the CFA/I/II/IV-STa_N12S_-dmLT or co-immunized with the CFA/I/II/IV MEFA and the 3xSTa_N12S_-dmLT toxoid fusion.

Mouse immunization groups^a^	Mean serum IgG titer (log_10_) ± stdev							
	anti-CFA/I	anti-CS1	anti-CS2	anti-CS3	anti-CS4/6	anti-CS5/6	anti-STa	anti-LT
CFA/I/II/IV- STa_N12S_-dmLT (n = 15)	2.63 ± 0.09	2.54 ± 0.04	2.44 ± 0.10	2.50 ± 0.09	2.58 ± 0.06	2.66 ± 0.03	2.50 ± 0.52	2.12 ± 0.30
CFA/I/II/IV and 3xSTa_N12S_-dmLT (n = 16)	2.69 ± 0.05	2.56 ± 0.05	2.53 ± 0.08	2.50 ± 0.06	2.59 ± 0.03	2.67 ± 0.05	2.88 ± 0.08	2.27 ± 0.13
p values^b^	0.04	0.33	0.01	0.97	0.58	0.58	0.01	0.10

^a^: Groups of mice immunized with CFA/I/II/IV-STa_N12S_-dmLT MEFA, or co-immunized with CFA/I/II/IV and 3xSTa_N12S_-dmLT proteins. Anti-CFA/I, anti-CS1, anti-CS2, anti-CS3, anti-CS4/CS5 and anti-CS5/CS6, and anti-STa and anti-LT IgG in serum sample of each immunized mouse was titrated by ELISAs using heat-extracted adhesin (500 ng adhesin per well of 2HB plates), STa conjugates (10 ng STa-ovalbumin conjugates per well of CoStar plates) and LT (100 ng LT per well of 2HB plates) as the coating antigen (in triplicates) and HRP-conjugated goat anti-mouse IgG (1:3300; Sigma) as the secondary antibodies. IgG titers (in log_10_) were expressed in means ± standard deviation.

^b^: p values were calculated by using a Student *t* test comparing mouse antibody titers in each group (15 mice immunized with CFA/I/II/IV-STa_N12S_-dmLT, 16 mice co-immunized with CFA/I/II/IV and 3xSTa_N12S_-dmLT).

There were no IgG antibodies specific to CFA/I, CFA/II, CFA/IV, STa or LT detected in the serum samples of the control mice or serum samples collected before the primary immunization.

### Mice immunized with CFA/I/II/IV-STa_N12S_-dmLT and mice co-immunized with CFA/I/II/IV and 3xSTa_N12S_-dmLT fusions developed similar or comparable levels of anti-CFA and antitoxin antibody responses

Serum samples of the mice immunized with CFA/I/II/IV-STa_N12S_-dmLT MEFA and the mice co-immunized with CFA/I/II/IV and 3xSTa_N12S_-dmLT developed similar titers of IgG antibodies to CS1, CS3, CS4/6, CS5/6 and LT (Table 2). Serum of the co-immunized mice (with CFA/I/II/IV and 3xSTa_N12S_-dmLT antigens) had greater titers of anti-CFA/I (2.69±0.05 vs 2.63±0.09; p = 0.04), anti-CS2 (2.53±0.08 vs 2.44±0.10; p = 0.01) and anti-STa (2.88±0.08 vs 2.50±0.52; p = 0.01) IgG antibodies, compared to the serum of the mice immunized with CFA/I/II/IV-STa_N12S_-dmLT MEFA.

### Serum samples of the immunized mice were shown to inhibit adherence of ETEC strains expressing CFA/I, CS3, CS4/CS6, CS5/CS6 or CS6 and *E*. *coli* strains expressing CS1 or CS2 to Caco-2 cells

Serum samples pooled from the mice co-immunized with CFA/I/II/IV and 3xSTa_N12S_-dmLT exhibited significant inhibition activities against adherence of H10407 (CFA/I^+^LT^+^STa^+^), E116 (CS3^+^LT^+^STa^+^), E106 (CS4^+^CS6^+^LT^+^STa^+^), UM75688 (CS5^+^CS6^+^LT^+^STa^+^) and 2423/ETP98066 (CS6^+^LT^+^STa^+^), and *E*. *coli* recombinant strains expressing CS1 adhesin or CS2 adhesins (Table 3). Serum sample pooled from the mice immunized with CFA/I/II/IV-STa_N12S_-dmLT showed significant adherence inhibition activities against all examined ETEC and *E*. *coli* strains except the recombinant *E*. *coli* strain expressing CS2.

**Table 3:** Results of *in vitro* antibody adherence inhibition assays^a^, using serum samples of mice immunized with CFA/I/II/IV-STa_N12S_-dmLT, co-administrated with CFA/I/II/IV MEFA and toxoid fusion 3xSTa_N12S_-dmLT, or the negative control mice. The number of ETEC or *E*. *coli* bacteria adhered to Caco-2 cells was used to indicate activity of anti-CFA antibodies against bacteria adherence.

Mouse serum^b^	CFA/I/II/IV-STa_N12S_-dmLT	CFA/I/II/IV + 3xSTa_N12S_-dmLT	control
Bacteria (CFUs)			
H10407; CFA/I, LT, STa (x10^4^)	14.4 ± 14.8 p^c^ = 0.0018	39.7 ± 9.6 p = 0.017	107.5 ± 9.2
THK38/pEU405; CS1 (x10^3^)	18.3 ± 7.7 p = 0.012	20.7 ± 11.6 p = 0.003	77 ± 7.1
DH5a/pEU588; CS2 (x10^3^)	13.5 ± 4.8 p = 0.28	7.5 ± 4.5 p = 0.017	16.5 ± 2.1
E116; CS3, LT, STa (x10^3^)	129 ± 49.9 p = 0.003	86 ± 49.6 p <0.001	235 ± 7.1
E106; CS4/CS6, LT, STa (x10^4^)	15.6 ± 3.3 p < 0.001	26.7 ± 3.9 p < 0.001	125 ± 0
UM 75688; CS5/CS6, LT, STa (x10^3^)	59.5 ± 27.3 p = 0.029	55 ± 35.2 p = 0.025	110 ± 14.1
ETP98066; CS6, LT, STa (x10^4^)	45.3 ± 28.6 p<0.001	53.3 ± 27.1 p<0.001	201.2 ± 29.5

^a^: ETEC field isolates and *E*. *coli* recombinant strains expressing CFA/I, CS1, CS2, CS3, CS4/CS6, CS5/CS6 and CS6 (3.5x10^6^ CFUs) were individually incubated with serum samples (20 μl) pooled from mice in the group immunized with CFA/I/II/IV-STa_N12S_-dmLT, co-immunized with the CFA/I/II/IV MEFA and 3xSTa_N12S_-dmLT, or the control mice on a shaker (50 rpm) for 1 hour at room temperature. The serum-bacteria mixture was added to Caco-2 cells (7x10^5^ cells; 1 ml final volume) and incubated in a CO_2_ incubator for 1 h. After washing off non-adherent bacteria, ETEC or *E*. *coli* bacteria adhered to Caco-2 cells (in 1 ml PBS) were serial diluted, plated, cultured overnight, and counted (CFUs).

^b^: serum samples pooled from mice of the group immunized the CFA/I/II/IV-STa_N12S_-dmLT, the group co-immunized with CFA/I/II/IV and 3xSTa_N12S_-dmLT, or the control group. These serum samples were used in the antibody adherence inhibition assay.

^c^: p values were calculated by using a Student *t* test comparing numbers of ETEC or *E*. *coli* bacteria adhered to the Caco-2 cells incubated with mouse serum of each immunization group vs. bacteria adherent to the cells treated with serum of the control group.

### Antibodies in serum samples of the immunized mice showed neutralizing activities against STa toxin and CT *in vitro*

**Fig 3:**
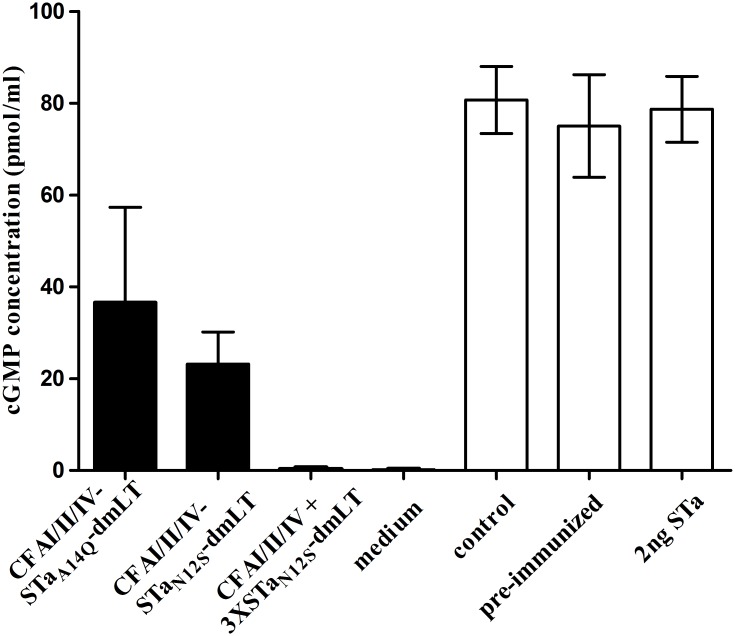
Mouse serum *in vitro* antibody neutralization activity against STa toxin. Intracellular cyclic GMP concentration (pmol/ml) in T-84 cells incubated with STa toxin and mouse serum was measured with an EIA cGMP ELISA kit (Assay Design) and was used to indicate anti-STa antibody neutralizing activity. STa toxin elevates intracellular cGMP in T-84 cells, whereas neutralizing anti-STa antibodies neutralize the toxin and prevent STa from stimulating cGMP, thus a lower cGMP concentration indicates a stronger neutralization activity of anti-STa antibodies. The serum sample (30 μl; in a final dilution of 1:33.3) pooled from each group of mice immunized with CFA/I/II/IV-STa_A14Q_-dmLT or CFA/I/II/IV-STa_N12S_-dmLT, co-immunized with CFA/I/II/IV and 3xSTa_N12S_-dmLT, the control group, or the serum sample collected prior to immunization was incubated with STa toxin (2 ng, in 150 μl cell culture medium) for 30 min at room temperature, and the serum-toxin mixture was added to T-84 cells (1 ml of final volume with cell culture medium). Intracellular cGMP concentration in T-84 cells was measured after 1 hour incubation at a CO_2_ incubator, with the mean cGMP and standard deviation (from four to six replicates) of each group indicated as columns and bars. The cGMP levels in T-84 cells cultured with cell culture medium alone (without STa toxin or serum; no STa toxicity) or with STa toxin in culture medium (without serum; STa toxicity) were used as controls.

Pooled serum samples from mice immunized with CFA/I/II/IV-STa_A14Q_-dmLT or CFA/I/II/IV-STa_N12S_-dmLT, or co-immunized with CFA/I/II/IV MEFA and 3xSTa_N12S_-dmLT showed neutralization activity against both STa and CT. Intracellular cGMP levels in T-84 cells incubated with 2 ng STa toxin and the serum of the mice immunized with CFA/I/II/IV-STa_A14Q_-dmLT, CFA/I/II/IV-STa_N12S_-dmLT, or CFA/I/II/IV combined with 3xSTa_N12S_-dmLT were 36.7±20.7, 23.2±7.0, 0.31±0.42 (pmol/ml), respectively (Fig. 3). These cGMP levels were significantly lower than the cGMP levels in T-84 cells incubated with 2 ng STa toxin and serum of the control mice (80.7±7.3; p = 0.007, <0.001, <0.001 in Student’s *t*-test, and p<0.01, <0.001, <0.001 in Mood’s Median Test) or the serum collected from pre-immunized mice (75.1±11.2; p = 0.03, <0.001, <0.001 in Student’s *t*-test, and p<0.01, <0.001, <0.001 in Mood’s Median Test). The cGMP level in T-84 cells incubated with 2 ng STa toxin alone was 78.7±7.2 pmol/ml.

**Fig 4:**
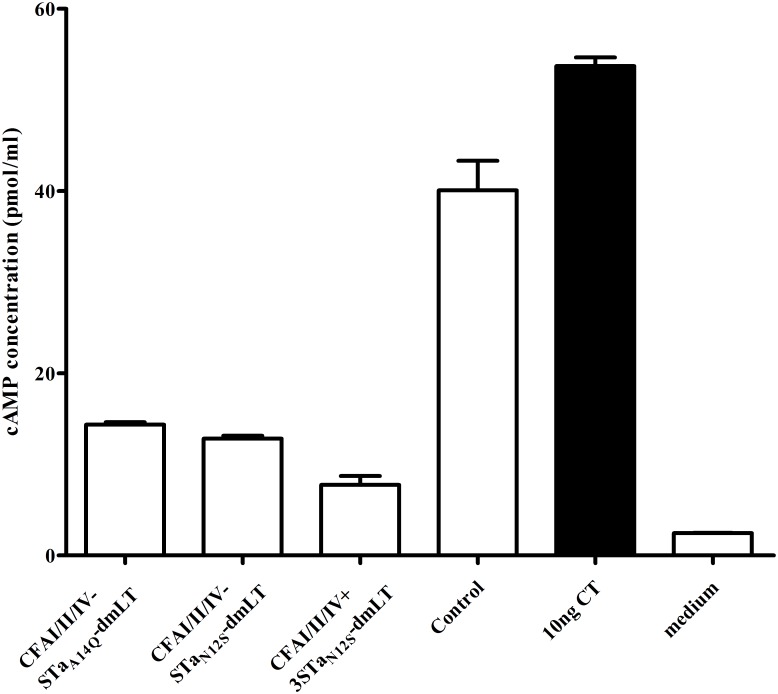
Mouse serum *in vitro* antibody neutralization activity against cholera toxin (CT). Intracellular cAMP concentrations (pmol/ml) in T-84 cells measured with an EIA cAMP ELISA kit (Assay Design) were used to indicate anti-LT antibody neutralizing activity. Neutralizing anti-LT antibodies neutralize CT toxin and prevent CT from stimulating cAMP in T-84 cells, thus resulting in a lower intracellular cAMP level. The serum sample (30 μl; in a final dilution of 1:33.3) pooled from each group of mice immunized with CFA/I/II/IV-STa_A14Q_-dmLT or CFA/I/II/IV-STa_N12S_-dmLT, co-immunized with CFA/I/II/IV and 3xSTa_N12S_-dmLT, or the control group was incubated with CT toxin (10 ng, in 150 μl cell culture medium) for 30 min at room temperature, and the serum-toxin mixture was added to T-84 cells (1 ml of final volume with cell culture medium). Intracellular cAMP concentration (pmole/ml) in T-84 cells was measured after 3 hour incubation at a CO_2_ incubator, with the mean cAMP and standard deviation (from four to six replicates) of each group indicated as columns and bars. The cAMP levels in T-84 cells incubated with cell culture medium alone (without CT or serum; no CT toxicity) or with CT in culture medium (without serum; CT toxicity) were also measured as references.

The cAMP levels in T-84 cells incubated with CT (10 ng) and the pooled serum sample of mice immunized with CFA/I/II/IV-STa_A14Q_-dmLT, CFA/I/II/IV-STa_N12S_-dmLT, or CFA/I/II/IV co-immunized with 3xSTa_N12S_-dmLT were 14.4±0.51, 12.8±0.76, and 7.7±2.4 pmol/ml, respectively (Fig. 4). These cAMP levels were significantly lower than those in cells incubated with the toxin alone (53.7±1.3; p<0.01 in Student’s *t*-test and Mood’s Median Test) or with the toxin and the serum sample of the control mice (40.1±6.5; p<0.01 in Student’s *t*-test and Mood’s Median Test).

Results from this study showed that CFA/I/II/IV-STa_A14Q_-dmLT and CFA/I/II/IV-STa_N12S_-dmLT MEFAs retained anti-CFA and antitoxin antigenicity as both induced antibody responses to seven heterogeneous ETEC CFA adhesins and LT and STa toxins. That suggested potential application of CFA/I/II/IV-STa_-toxoid_-dmLT MEFAs in development of broadly protective ETEC vaccines. Vaccines inducing anti-CFA antibodies to broadly prevent ETEC adherence and colonization and also antitoxin antibodies to neutralize both LT and STa toxins are expected to be broadly effective against ETEC diarrhea. To develop a vaccine carrying representative antigens of all or even a majority of the 23 characterized CFA adhesins and also two toxins is an unreachable goal currently. But it appears feasible to develop a vaccine protecting against ETEC strains that cause a majority of clinical cases including moderate to severe cases. Vaccine candidates currently under development carry antigens of up to six CFA adhesins and CT-B or LT-B subunit, but they do not induce anti-STa immunity and also require a high oral delivery dose with excessive amount of somatic antigen. These vaccine candidates would have to include additional strains to deliver additional CFAs and STa antigens in order to induce broader anti-CFA and antitoxin antibody responses. But inclusion of additional strains would require an even higher oral dose leading to potentially greater side effects.

A vaccine with fewer strains, or ideally a single strain, to express multiple CFA adhesins and LT and STa toxins (or antigenic elements of these CFA adhesins and toxins) could overcome the difficulties encountered by the cocktail vaccine candidates. Recently, we demonstrated that a single CFA/I/II/IV MEFA carrying epitopes of seven CFA adhesins [CFA/I, CFA/II (CS1, CS2, CS3) and CFA/IV (CS4, CS5, CS6)] induced antibodies broadly inhibiting adherence of ETEC and *E*. *coli* strains expressing these seven CFA adhesins [39]. Protection against these seven CFA adhesins can potentially protect against ETEC strains associated with 70% to 80% of ETEC diarrhea cases [23,40] and also the moderate to severe cases [46]. Unlike cocktail vaccine candidates, this CFA/I/II/IV MEFA was expressed as a single protein by a single *E*. *coli* strain, and for the first time induced antibody responses in mice protecting against all seven CFA adhesins. Products developed from this CFA/I/II/IV MEFA can be formulated and administrated at a regular or lower dose, thus will eliminate the risk of adverse effects associated with the cocktail vaccine candidates. In addition, using a peptide or epitopes instead of an entire adhesin as the antigen may have advantage. A peptide from an adhesin subunit can be more effective in inducing adhesin-specific antibody responses. Experimental vaccines carrying intact F18 fimbriae, physically purified (as a subunit vaccine) or expressed by a live *E*. *coli* strain (as a live vaccine), were unable to induce effective antibody responses to protect pigs against F18-fimbrial ETEC challenge [47,48]; however, a small peptide from the minor subunit of the F18 fimbrial adhesin was shown to induce strongly protective antibodies against this F18 adhesin [44,49]. Data from this study showed that mice immunized with the CFA/I/II/IV MEFA (co-administrated with the toxoid fusion) or the CFA/I/II/IV-STa_-toxoid_-dmLT that carried a part of the CFA/I major subunit peptide developed a similar anti-CFA/I IgG titer as mice immunized with CFA/I fimbriae.

We reported recently that a STa toxoid, such as STa_A14Q_ or STa_N12S_, can be genetically fused to a monomeric LT toxoid peptide and the resultant LT-STa toxoid fusions elicited antibodies neutralizing STa and LT toxins [37,38]. Results from the toxoid fusion and the CFA/I/II/IV MEFA studies led us to believe that a single CFA/I/II/IV-STa_-toxoid_-dmLT MEFA could induce broad anti-CFA and antitoxin antibody responses and can be potentially used for development of a broadly protective ETEC vaccine. Data from the present study showed mice immunized with CFA/I/II/IV-STa_A14Q_-dmLT or CFA/I/II/IV-STa_N12S_-dmLT developed antibody responses to all seven adhesins and both toxins. That indicated that the CFA/I/II/IV-STa_-toxoid_-dmLT MEFA retained antigenicity of individual CFA adhesins and toxins, and suggested potential application of CFA/I/II/IV-STa_-toxoid_-dmLT MEFA in ETEC vaccine development.

Data from this study showed that mice immunized with the CFA/I/II/IV-STa_N12S_-dmLT developed greater titer of anti-STa IgG antibodies (2.50±0.52; in log_10_) than mice immunized with CFA/I/II/IV-STa_A14Q_-dmLT (2.40±0.75); also, serum antibodies induced by CFA/I/II/IV-STa_N12S_-dmLT exhibited greater neutralizing activity against STa enterotoxicity (Fig. 3). That confirmed STa_N12S_ is the preferred toxoid for fusions to induce anti-STa antibody response. However, it was noted that IgG antibody titers to all CFA adhesins and LT in serum of the mice immunized with the CFA/I/II/IV-STa_N12S_-dmLT were found systematically lower than those in the serum of mice immunized with CFA/I/II/IV-STa_A14Q_-dmLT. Given the fact that the only difference between these two MEFA antigens was the different STa toxoid, these two MEFA antigens should have induced similar titers of antibody responses to the CFAs and LT. Future studies will be needed to explore whether different STa toxoids alter antigenic structure of CFA/I/II/IV-STa_-toxoid_-dmLT MEFAs.

Since CFA/I/II/IV-STa_N12S_-dmLT MEFA induced antibodies with greater neutralizing activity against STa (compared to CFA/I/II/IV-STa_A14Q_-dmLT MEFA) and also significant adherence inhibition against ETEC or *E*. *coli* strains expressing six of the seven adhesins, this MEFA was evaluated for potential application in multivalent vaccine development. By comparatively examining antigen-specific antibody responses in mice immunized with the single CFA/I/II/IV-STa_N12S_-dmLT MEFA and in mice immunized with a combination of CFA/I/II/IV MEFA and toxoid fusion 3xSTa_N12S_-dmLT, we found that mice in the two immunization groups developed similar levels of anti-CS1, -CS3, -CS4/6 and anti-CS5/6, and anti-LT IgG antibody responses (Table 2). That suggested that the fusion process did not compromise antigenicity of a majority of the individual antigenic components. It was observed, however, that mice co-immunized with CFA/I/II/IV MEFA and 3xSTa_N12S_-dmLT fusion developed moderately greater titers of anti-CFA/I (2.69±0.05 vs 2.63±0.9; p = 0.04), anti-CS2 (2.53±0.08 vs 2.44±0.10; p = 0.01) and anti-STa (2.88±0.08 vs 2.50±0.52; p = 0.01) serum IgG antibodies. The negative effect on anti-CFA/I and anti-CS2 immunogenicity could be resulted from alteration of the CFA/I and CS2 epitope antigenic topology in the CFA/I/II/IV-STa_N12S_-dmLT MEFA, since the CFA/I epitope (^159^SGVVSLVMT^167^) and the CS2 epitope were located at the C-terminus of the CFA/I/II/IV MEFA to which the toxoid fusion was fused. Future protein structure studies may help to reveal if any structural alteration occurred. Since the anti-adhesin antibodies induced by the CFA/I/II/IV-STa_N12S_-dmLT MEFA were able to significantly inhibit adherence of ETEC H10407 strain but not against CS2 strain (Table 3), we may only need to optimize the construction to enhance anti-CS2 immunogenicity. To relocate the CS2 epitope toward the N-terminus or to extend the linker between CFA/I/II/IV MEFA and the toxoid fusion may improve CS2 epitope presentation and anti-CS2 immunogenicity.

Data from the study showed a lower anti-STa IgG antibody response in mice immunized with CFA/I/II/IV-STa_N12S_-dmLT MEFA compared to the mice co-administrated with the CFA/I/II/IV and 3xSTa_N12S_-dmLT (Table 2). That was not surprising because only two copies of STa_N12S_ were carried by CFA/I/II/IV-STa_N12S_-dmLT MEFA (versus three STa_N12S_ toxoids were carried by the 3xSTa_N12S_-dmLT used for co-administration). Additional copies of a STa toxoid in a LT-STa toxoid fusion were shown to enhance anti-STa antigenicity [37]. That also explained that only serum sample from the co-administrated mice was able to completely neutralized 2 ng STa (Fig. 3). Had CFA/I/II/IV-STa_N12S_-dmLT MEFA carried three copies of STa_N12S_, we would have observed a similar level of anti-STa antibody response and antibody neutralization activity against STa from the serum of the immunized mice. Future studies to modify the CFA/I/II/IV-STa_N12S_-dmLT MEFA to carry three copies of STa_N12S_, and to examine whether it induces the same level of anti-STa antibody response and more importantly antibodies with similar neutralizing activity against STa as the combination of 3xSTa_N12S_-tmLT and CFA/I/II/IV will be informative.

Data from the study also showed that serum of mice co-immunized with CFA/I/II/IV MEFA and 3xSTa_N12S_-dmLT showed greater neutralization activity against CT (Fig. 4). In contrast to CFA/I/II/IV-STa_A14Q_-dmLT or CFA/I/II/IV-STa_N12S_-dmLT MEFA which contained the last 109 amino acids of LT-A1, the co-administrated toxoid fusion 3xSTa_N12S_-dmLT carried the entire LT-A1 peptide. That suggested the antibody response to the LT-A1 peptide could play an important role in neutralizing against LT or CT toxin.

Although results from the current study suggested that a fusion of the CFA/I/II/IV MEFA and the toxoid fusion did not significantly alter antigenicity of the carried adhesin and toxin antigenic components, the combination of the CFA/I/II/IV MEFA and toxoid fusion 3xSTa_N12S_-dmLT seemed the better antigens for future ETEC vaccine development as these two antigens induced greater titers of anti-STa, anti-CFA/I and anti-CS2 antibodies than the single CFA/I/II/IV-STa_N12S_-dmLT MEFA. But a vaccine product derived from a single antigen or a single strain decreases complexity and cost in product manufacture. If the CFA/I/II/IV-STa_N12S_-dmLT MEFA, with modification to carry an additional copy of STa_N12S_ and to have anti-CS2 antigenicity enhanced, is safe and induces equivalent antibody responses to all seven adhesins and both toxins as the combination of CFA/I/II/IV and 3xSTa_N12S_-dmLT, this modified CFA/I/II/IV-STa_N12S_-dmLT MEFA should be the preferred antigen for ETEC vaccine development.

It needs to point out that only intraperitoneal immunization route was used to examine immunogenicity of the CFA/I/II/IV-STa_-toxoid_-dmLT MEFAs in this study. Future studies using intradermal, intramuscular, subcutaneous or even oral route, and perhaps with different adjuvants such as dmLT which is also an antigen to further induce neutralizing anti-LT antibodies, will help us to characterize better the immunogenicity of a modified CFA/I/II/IV-STa_N12S_-dmLT MEFA. In addition, fusion protein structure, protein integrity and stability, and more importantly safety of this CFA/I/II/IV-STa_N12S_-dmLT MEFA, as well as the CFA/I/II/IV MEFA and the 3xSTa_N12S_-dmLT (for co-administration), would have to be thoroughly characterized before it can be assessed for ETEC vaccine development. Furthermore, animal models including a piglet challenge model and a rabbit colonization model, and perhaps the modified RITARD (removable intestine tie adult rabbit diarrhea) model, will be needed in preclinical studies to evaluate protective efficacy of the induced anti-CFA and antitoxin immunity against ETEC diarrhea prior to human volunteer studies or field trials. Additionally, despite of results from a recent study indicated that anti-STa monoclonal and polyclonal antibodies had no reactivity with guanylin and a very low reactivity with uroguanylin [50], anti-STa antibodies induced by the toxoid fusion or CFA-toxoid MEFAs would need to be evaluated for cross reactivity with guanylin and uroguanylin. This study, nevertheless, demonstrated that epitopes from multiple CFA adhesins and antigens from both toxins can be fused together as a single MEFA protein to induce broadly protective antibody responses, and suggested potential application of MEFA for effective ETEC vaccine development. In addition, as plasticity in MEFA construction allows inclusion of additional antigens for even broader protection, this MEFA approach perhaps can be generally used in effective vaccine development against diseases caused by other immunologically heterogeneous pathogenic strains or isolates.

## Conclusions

Results from this study indicated CFA/I/II/IV-STa_-toxoid_-dmLT MEFA or CFA/I/II/IV MEFA combined with 3xSTa_N12S_-dmLT induced broadly protective anti-CFA and antitoxin immunity, and suggested their potential application in broadly effective ETEC vaccine development. This MEFA strategy may be generally used in multivalent vaccine development.
